# Flexible and Stretchable Pressure Sensors: From Basic Principles to State-of-the-Art Applications

**DOI:** 10.3390/mi14081638

**Published:** 2023-08-20

**Authors:** Thara Seesaard, Chatchawal Wongchoosuk

**Affiliations:** 1 Department of Physics, Faculty of Science and Technology, Kanchanaburi Rajabhat University, Kanchanaburi 71190, Thailand; thara.seesaard@kru.ac.th; 2 Department of Physics, Faculty of Science, Kasetsart University, Bangkok 10900, Thailand

**Keywords:** pressure sensor, piezoresistivity, capacitance, piezoelectricity, flexible electronic

## Abstract

Flexible and stretchable electronics have emerged as highly promising technologies for the next generation of electronic devices. These advancements offer numerous advantages, such as flexibility, biocompatibility, bio-integrated circuits, and light weight, enabling new possibilities in diverse applications, including e-textiles, smart lenses, healthcare technologies, smart manufacturing, consumer electronics, and smart wearable devices. In recent years, significant attention has been devoted to flexible and stretchable pressure sensors due to their potential integration with medical and healthcare devices for monitoring human activity and biological signals, such as heartbeat, respiratory rate, blood pressure, blood oxygen saturation, and muscle activity. This review comprehensively covers all aspects of recent developments in flexible and stretchable pressure sensors. It encompasses fundamental principles, force/pressure-sensitive materials, fabrication techniques for low-cost and high-performance pressure sensors, investigations of sensing mechanisms (piezoresistivity, capacitance, piezoelectricity), and state-of-the-art applications.

## 1. Introduction

In recent years, the field of wearable electronics has experienced remarkable advancements driven by the increasing demand for portable and seamlessly integrated devices in everyday life. Among the essential components in wearable technology, pressure sensors play a pivotal role in monitoring physiological and environmental parameters [1]. However, traditional pressure sensors, typically constructed from rigid materials, present challenges in terms of comfort, wearability, and conformity to curved surfaces. These limitations have sparked the exploration of flexible and stretchable pressure sensors as a viable alternative. The driving force behind these research studies is the need to overcome the constraints of traditional pressure sensors and fully unleash the potential of wearable technology. Flexible and stretchable pressure sensors offer numerous advantages over their rigid counterparts. Firstly, their ability to conform to irregular and curved surfaces, including the human body, allows for the comfortable and unobtrusive monitoring of vital signs, such as blood pressure [2], pulse [3], and respiration rate [4]. This feature is particularly valuable in healthcare applications where continuous non-invasive monitoring is crucial for early detection and management of various medical conditions. Secondly, the stretchability of these sensors enables them to endure deformation and mechanical strain without compromising their functionality. This attribute is highly desirable in applications involving human motion, such as sports monitoring [5], rehabilitation [6], and virtual reality interfaces [7]. The ability of pressure sensors to stretch and bend with the body ensures accurate and reliable measurements, even during dynamic activities. Furthermore, the development of flexible and stretchable pressure sensors presents new possibilities for human–machine interfaces and robotics [8]. Integration of these sensors into prosthetic limbs and exoskeletons can provide users with enhanced proprioception and natural interaction with their robotic counterparts [9]. Additionally, the integration of pressure sensors into smart clothing and textiles allows for intuitive and gesture-based control of electronic devices and appliances [10]. The research on flexible and stretchable pressure sensors is also motivated by the growing interest in the Internet of Things (IoT) and smart systems [11]. Embedding pressure-sensing capabilities into everyday objects, such as furniture [12], automotive interiors [13], and industrial equipment [14], enables the creation of intelligent environments that can adapt to human needs and preferences. These sensors can provide valuable data for monitoring structural integrity, optimizing resource allocation, and enhancing user comfort and safety. Despite significant progress in the development of flexible and stretchable pressure sensors, several challenges persist. Achieving high sensitivity, durability, and reliability while maintaining mechanical flexibility poses engineering and material science challenges. The fabrication processes need to be scalable, cost-effective, and compatible with different substrates and integration platforms. Additionally, ensuring biocompatibility and safety for applications involving direct contact with the human body is of utmost importance.

**Figure 1:**
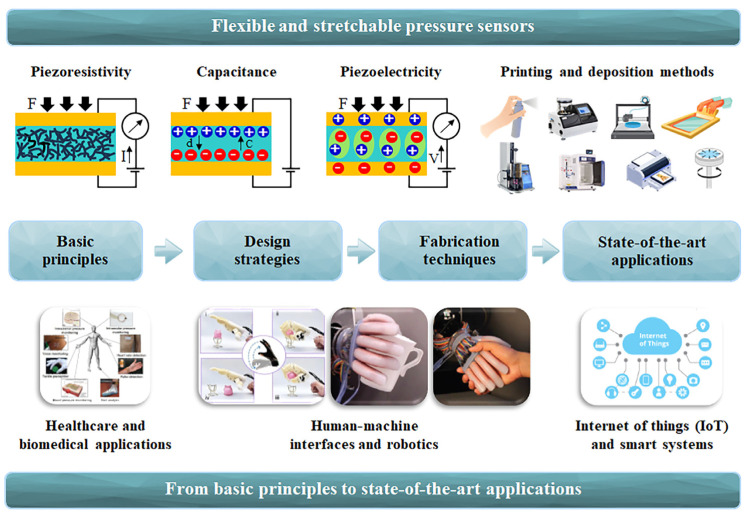
An overall review of research on flexible and stretchable pressure sensors from basic principles to state-of-the-art applications.

Considering the significant challenges faced and the potential impact of flexible and stretchable pressure sensors in diverse fields, this comprehensive review aims to provide a comprehensive and in-depth understanding of the fundamental principles, design strategies, fabrication techniques, and state-of-the-art applications associated with these flexible and stretchable pressure sensors. It encompasses four sections: Section 1 focuses on the basic principles of the operation of these sensors. Section 2 delves into the designed strategies employed in the development of flexible and stretchable pressure sensors, including sensor architecture, materials selection, and optimization techniques. By examining the design strategies, this section aims to highlight effective approaches for enhancing sensor performance and functionality. Section 3 presents the fabrication techniques, such as printing, lithography, and assembly methods, that enable the mass production of these sensors while ensuring their mechanical flexibility and high performance. Section 4 demonstrates the state-of-the-art applications, including healthcare, human–machine interfaces, robotics, and IoT systems, that demonstrate the potential of these flexible and stretchable pressure sensors in revolutionizing diverse fields and fostering innovation. The overview of this review is depicted in Figure 1.

## 2. Basic Principles of Flexible and Stretchable Pressure Sensors

### 2.1. Sensing Mechanisms

Flexible and stretchable pressure sensors employ various sensing mechanisms to detect and measure the pressure. The choice of sensing mechanism depends on the specific design requirements, desired sensing range, sensitivity, and accuracy. Here, we discuss three commonly used sensing mechanisms in flexible and stretchable pressure sensors: resistive sensing, capacitive sensing, piezoelectric sensing.

#### 2.1.1. Resistive Sensing

**Figure 2:**
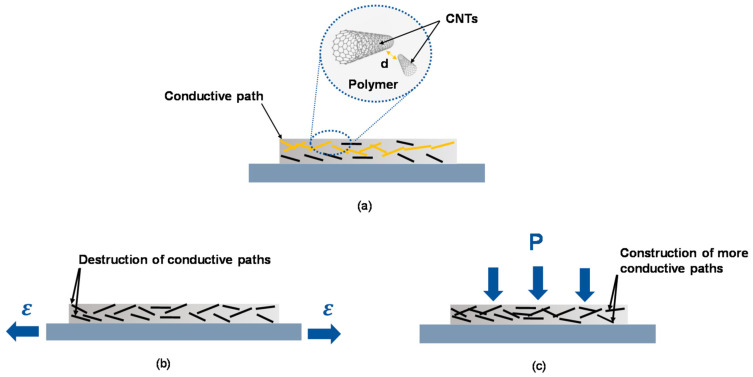
Resistive sensing mechanism of polymer/CNT-based flexible and stretchable pressure sensor: (**a**) no applied force/pressure, (**b**) under applied force, and (**c**) under applied pressure [22].

Resistive sensing is a fundamental mechanism employed in flexible and stretchable pressure sensors to detect and measure pressure-induced deformation. The basic principle of resistive sensing involves the measurement of changes in electrical resistance resulting from the applied pressure. Flexible and stretchable pressure sensors utilizing resistive sensing typically consist of a conductive material embedded in a flexible matrix. The conductive materials can be carbon nanotubes (CNTs) [15], graphene [16,17], conductive polymers [18], ZnO nanoparticles [19], silver oxide nanoparticles [20] or lead oxide nanoparticles [21] with suitable electrical properties. When pressure is applied to the sensor, the conductive elements deform to alter the electrical resistance/conductivity. The resistive sensing mechanism can be explained using polymer/CNT-based pressure sensors, such as the example [22] shown in Figure 2. The polymer acts as the host material while CNTs work as conductive paths. When pressure is applied to the sensor, the deformation of the substrate causes a change in the alignment or the separation of gap variation (d) between adjacent CNTs. This causes the effects of the charge carrier transfer of the conducting path of CNTs into the polymer matrix. Thus, the resistance of the polymer/CNT sensor depends on the magnitude and direction of applied force/pressure.

The advantage of resistive sensing in flexible and stretchable pressure sensors is its simplicity and compatibility with a wide range of materials and fabrication techniques. It allows for the integration of sensing elements into flexible substrates and enables conformability to curved surfaces, including the human body [23]. Additionally, resistive sensing provides a direct electrical output, which can be easily interfaced with electronic circuits for signal processing and data analysis. However, there are certain challenges associated with resistive sensing in flexible and stretchable pressure sensors. These include the need for stable and uniform conductive networks, the development of reliable and durable electrode–substrate interfaces, and the mitigation of hysteresis effects caused by material fatigue or creep. These challenges still require a careful material selection, optimized sensor designs, and robust fabrication processes.

#### 2.1.2. Capacitive Sensing

**Figure 3:**
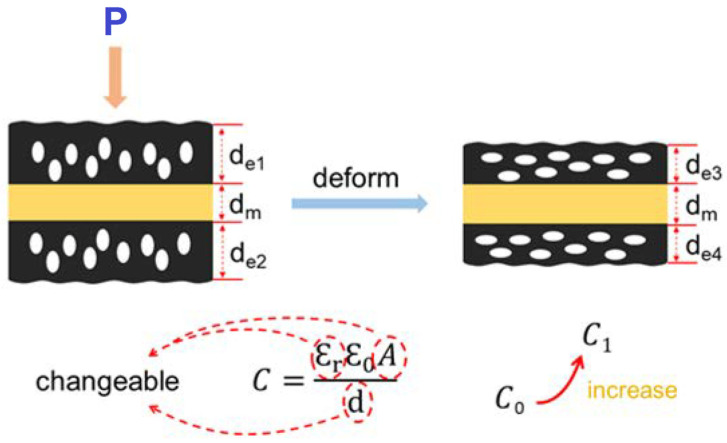
Capacitive sensing mechanism of flexible pressure sensor based on porous electrodes [26].

The basic principle of capacitive sensing involves the measurement of variations in capacitance resulting from the applied pressures. Flexible and stretchable pressure sensors employing capacitive sensing typically consist of two conductive layers separated by a dielectric layer. The conductive layers can be made of metals [24] or conductive polymers [25] while the dielectric layer is often an elastomeric material with a high dielectric constant [26]. When pressure is applied to the sensor, the deformation of the elastomeric layer leads to a change in the separation distance between the conductive layers, thereby altering the capacitance. To elucidate the fundamental tenets of capacitive sensing, we exemplify with a capacitive pressure sensor based on porous electrodes [22], as shown in Figure 3. In this type of sensor, two conductive layers are patterned on flexible substrates, and a dielectric layer is placed between them. The conductive layers can be designed as electrodes or interdigitated structures to maximize the sensing area. When pressure is applied to the sensor, the elastomer layer compresses, reducing the separation distance between the conductive layers. This change increases/decreases in capacitance according to the general equation C = εA/d, where C is the capacitance, ε is the permittivity of the dielectric, A is the overlapping area of the conductive layers, and d is the separation distance [27].

The advantage of capacitive sensing in flexible and stretchable pressure sensors lies in its high sensitivity and compatibility with flexible substrates. Capacitive sensors can exhibit excellent mechanical stretchability and conformability, enabling their integration into various applications, including wearable devices and robotic skins. However, capacitive sensing also presents challenges that need to be addressed. These challenges include minimizing parasitic capacitance and resistance effects, optimizing the design and fabrication of the conductive layers and dielectric materials, and mitigating hysteresis and drift caused by environmental factors.

#### 2.1.3. Piezoelectric Sensing

**Figure 4:**
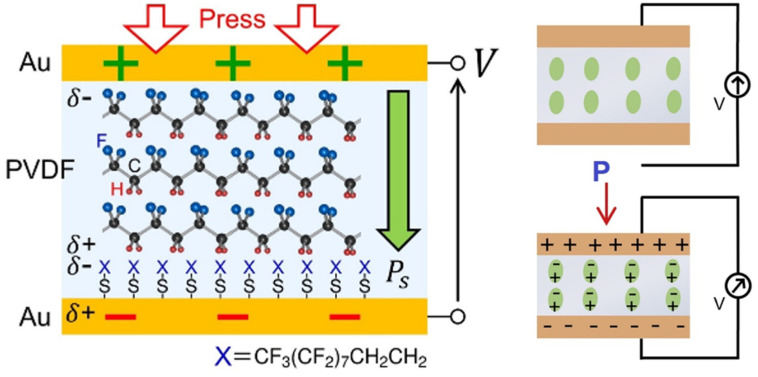
Piezoelectric sensing mechanism of pressure sensor based on PVDF sensing material. (Reproduced with permission from Elsevier [28,31]).

Piezoelectric sensing relies on the use of materials that can generate an electrical charge in response to mechanical stress or pressure. Piezoelectric materials exhibit a property known as the piezoelectric effect, which allows them to convert mechanical energy into electrical energy. When pressure is applied to a piezoelectric material, it deforms and induces an electrical charge or voltage across its surface, as shown in Figure 4. This charge or voltage can be measured and correlated with the applied pressure. In the context of flexible and stretchable pressure sensors, piezoelectric materials are integrated into the sensor structure to capture pressure-induced deformations and generate electrical signals. For example, polyvinylidene fluoride (PVDF) is a common piezoelectric material used in flexible and stretchable pressure sensors [28]. A thin film or sheet of PVDF is incorporated into the sensor structure, as displayed in Figure 4. The PVDF is typically polarized and owns a permanent dipole moment [29,30]. When pressure is applied to the sensor, the PVDF deforms, causing a change in its dipole moment and generating an electrical charge across its surface. The measurement of the electrical charge or voltage generated by the piezoelectric material is typically achieved by connecting the PVDF to electrodes and utilizing appropriate circuitry. The resulting electrical signal can be correlated with the applied pressure using calibration curves or mathematical models.

The advantage of piezoelectric sensing in flexible and stretchable pressure sensors is its direct conversion of mechanical energy into electrical signals, allowing for immediate and sensitive pressure detection. Additionally, piezoelectric sensors exhibit a broad sensing range and fast response times, making them suitable for dynamic pressure measurements. However, there are certain challenges associated with piezoelectric sensing in flexible and stretchable pressure sensors. They include material selection, ensuring uniformity and stability of the piezoelectric film or structure, and optimize the sensor design to enhance sensitivity and minimize interference from external factors.

The comparison of the advantages and disadvantages of pressure-sensing properties in flexible and stretchable pressure sensors for the five main properties, including sensitivity, range of measurement, mechanical durability, fabrication complexity, and power consumption, of three different mechanisms is summarized in Table 1.

### 2.2. Sensing Performance Metrics

Flexible and stretchable pressure sensors can be evaluated based on several performance metrics that assess their sensing capabilities. These metrics provide quantitative measures of the sensor’s accuracy, sensitivity, dynamic range, response time, and stability. The metrics are crucial for assessing the overall performance and suitability of flexible and stretchable pressure sensors for specific applications, as detailed below.

#### 2.2.1. Sensitivity

Sensitivity measures the ability of a pressure sensor to detect and quantify small changes in pressure. It is typically defined as the ratio of the change in output signal (e.g., voltage [48,49], capacitance [50,51,52], resistance [53,54]) to the corresponding change in applied pressure. For instance, the assessment of the sensitivity of a piezoresistive pressure sensor is defined by the following equation.

Sensitivity (S) = (ΔR/R_0_)/ΔP (1)

where ΔR/R_0_ = (R − R_0_)/R_0_ represents the quantified relative alteration in resistance as observed through measurements. R_0_ denotes the baseline resistance of the pressure sensor in the absence of any external pressure, while R signifies the resistance exhibited by the sensor upon being subjected to an external pressure acting upon its surface. This change in resistance relative to the baseline (ΔR/R_0_) functions as a dynamic representation of the applied pressure (ΔP). The slope inherent in this linear relationship defines the sensitivity of the pressure sensor [55]. A higher sensitivity indicates a greater responsiveness of the sensor to pressure variations.

#### 2.2.2. Dynamic Range

The dynamic range represents the span of pressures that a sensor can accurately measure. It is the difference between the minimum and maximum pressures that can be detected by the sensor while maintaining acceptable linearity and accuracy. A wider dynamic range allows the sensor to capture a broader range of pressure levels [56].

#### 2.2.3. Linearity

Linearity refers to the relationship between the measured output signal of the sensor and the actual applied pressure. An ideal sensor exhibits perfect linearity where the output signal is directly proportional to the applied pressure. Deviations from linearity can introduce measurement errors and affect the accuracy of the sensor [57].

#### 2.2.4. Response Time

The response time of a pressure sensor measures how quickly it can detect and provide an output signal in response to a change in pressure. It is typically defined as the time taken for the sensor’s output signal to reach a specified percentage (e.g., 90% or 95%) of its final value [58] after a pressure change. A shorter response time enables faster monitoring and real-time pressure measurements [59].

#### 2.2.5. Recovery Time

The recovery time refers to the period required for the sensor to return to its original state after experiencing a pressure change. Similar to response time, the recovery time can usually be determined as the time taken to reach 90% of the baseline value [60]. A shorter recovery time allows the sensor to respond quickly to new pressure inputs, ensuring accurate measurements, especially in dynamic applications [61].

#### 2.2.6. Hysteresis

Hysteresis refers to the phenomenon where the sensor’s output signal exhibits different responses for increasing and decreasing pressures, even if the applied pressure values are the same. Hysteresis can introduce measurement errors and affect the repeatability and accuracy of the sensor. A low hysteresis indicates better repeatability and stability of the sensor’s output [62].

#### 2.2.7. Drift

Drift refers to the long-term changes in the sensor’s output signal over time, even in the absence of applied pressure. It can be influenced by environmental factors such as temperature variations or aging of sensor materials. Minimizing drift is crucial for maintaining the accuracy and reliability of pressure measurements [63].

#### 2.2.8. Resolution

Resolution refers to the smallest increment of pressure that a sensor can detect and distinguish. It is determined by the noise level of the sensor’s output signal and defines the smallest detectable change in pressure. A higher resolution enables the sensor to provide more precise and detailed pressure measurements [64].

#### 2.2.9. Cross-Sensitivity

Cross-sensitivity quantifies how much a sensor’s output signal is influenced by factors beyond the applied pressure. For example, a pressure sensor designed for measuring physiological pressure may also be responsive to temperature changes or humidity. Minimizing cross-sensitivity ensures that the sensor’s output is primarily influenced by the intended pressure stimulus, thereby enhancing measurement accuracy [65].

To consider the sensing performance metrics with pressure sensor types, the piezoresistive pressure sensors exhibit moderate to high sensitivity to pressure changes, making them suitable for applications requiring accurate measurements. Their ability to cover a broad dynamic range allows versatility in different pressure conditions. Furthermore, piezoresistive sensors generally demonstrate good linearity, providing reliable and proportional output signals with pressure variations. They offer fast response times, enabling real-time monitoring in dynamic pressure scenarios. Additionally, these sensors have relatively quick recovery times, allowing for a rapid return to the initial state after pressure changes. However, drift in output readings over time usually occurs due to environmental factors or material aging. Despite these limitations, piezoresistive sensors offer high resolution for detecting small pressure changes accurately and are less prone to cross-sensitivity compared to other sensing technologies [66].

In the case of capacitive pressure sensors, they are well-known for their good sensitivity, particularly in detecting small pressure variations. Their broad dynamic range provides versatility for various applications. Capacitive sensors demonstrate good linearity, ensuring reliable and proportional output signals. With fast response times, they are well-suited for dynamic pressure measurements. Capacitive sensors also have relatively quick recovery times after pressure changes. However, these sensors may experience hysteresis, leading to some deviation in response during pressure cycling. Cross-sensitivity may be presented due to factors such as temperature or humidity variations. Drift in capacitance changes is typically minimal, ensuring stable and reliable performance over time [67].

For piezoelectric pressure sensors, they stand out for their high sensitivity to pressure changes, making them suitable for precise pressure measurements. Their broad dynamic range allows versatility in various pressure conditions. Piezoelectric sensors exhibit good linearity, ensuring accurate and proportional output signals. With fast response times, they are well-suited for dynamic pressure applications. Recovery times are quick, allowing for a rapid return to the initial state after pressure changes. However, piezoelectric sensors may exhibit hysteresis, leading to some deviation in response during pressure cycling [68]. Nevertheless, they demonstrate minimal drift in output readings, ensuring stable and reliable performance over time. High-resolution capabilities enable piezoelectric sensors to detect small pressure changes effectively. However, cross-sensitivity may exist due to external factors affecting piezoelectric material properties. The comparative analysis of piezoresistive, capacitive, and piezoelectric pressure sensors based on their sensing performance metrics is illustrated in Table 2.

## 3. Strategies for the Design of Flexible and Stretchable Pressure Sensors

Design strategies for flexible and stretchable pressure sensors revolve around incorporating materials and structures that enable conformability, durability, and enhanced sensing capabilities. Utilizing stretchable materials, such as fabric, elastomers, conductive polymers, or nanomaterials, allows the sensors to deform without compromising performance. Innovative structural designs, such as serpentine patterns or interdigitated electrodes on flexible structures like fabric [69], as well as pore structures in foam [70], help to maintain electrical conductivity during stretching. Additionally, the integration of microfabrication techniques enables the creation of sensor arrays for spatial pressure mapping. Furthermore, the development of hybrid sensors combining different transduction mechanisms, such as piezoresistive and capacitive sensing, offers enhanced sensitivity and reduced cross-sensitivity. These design approaches play a crucial role in advancing flexible and stretchable pressure sensors, making them suitable for a wide range of applications, including wearable devices and health monitoring systems.

### 3.1. Material Selection

#### 3.1.1. Elastomers

Designing flexible and stretchable pressure sensors involves careful consideration of various factors, with material selection being of paramount importance. Elastomers play a crucial role in these sensors due to their unique mechanical properties that enable deformability and stretchability [71]. In this analysis, we will explore the design strategies related to elastomer selection in flexible and stretchable pressure sensors.

- Mechanical flexibility and stretchability: Elastomers exhibit high elasticity, allowing them to conform to curved surfaces and withstand stretching and bending during sensor operation [72]. Designers select elastomers with high elongation and low stiffness to ensure that the sensor can withstand mechanical strain and deformation without compromising its functionality or durability.
- Sensitivity and responsiveness: The choice of elastomer impacts the sensor’s sensitivity to pressure variations, with high-sensitivity elastomers translating pressure-induced deformations into measurable signals. Design strategies involve selecting elastomers that exhibit a significant change in their mechanical properties (e.g., modulus, conductivity) in response to pressure [73]. This change can be utilized for accurate pressure detection and measurement.
- Conformability and conformal contact: Elastomers offer excellent conformability, allowing the sensor to adhere and conform to irregular and curved surfaces, including the human body or complex objects [74]. Designers choose elastomers that can maintain conformal contact with the target surface, ensuring reliable and accurate pressure measurements across various application scenarios [75].
- Biocompatibility and skin contact: In applications involving direct contact with the human body, such as healthcare or wearable devices, biocompatibility is crucial [76]. Design strategies focus on selecting elastomers that are biocompatible, hypoallergenic, and safe for prolonged skin contact [77]. They should not cause irritation or adverse reactions.
- Material compatibility and integration: Elastomers must be compatible with other components of the sensor, such as electrodes [78], interconnects, and encapsulation materials, to ensure proper integration and functionality [79]. Design strategies involve considering the compatibility of elastomers with fabrication techniques and processes used in sensor manufacturing, such as printing, molding, or deposition methods.
- Environmental stability: Elastomers should exhibit stability and reliability under environmental conditions encountered during sensor operation, including temperature variations, humidity, and exposure to chemicals or solvents. Designers consider the elastomer’s resistance to environmental factors to ensure long-term stability and performance of the sensor [80].
- Scalability: Design strategies aim to select elastomers that are readily available and compatible with scalable fabrication processes [81]. This ensures the feasibility of mass production and commercialization of flexible and stretchable pressure sensors.

#### 3.1.2. Conductive Materials

Designing flexible and stretchable pressure sensors requires careful consideration of conductive materials that play a crucial role in enabling the sensors’ sensing and signal transmission capabilities. This analysis explores the design strategies related to conductive material selection in flexible and stretchable pressure sensors.

- Electrical conductivity: Conductive materials with high electrical conductivity are preferred for the efficient transmission of electrical signals [82]. Design strategies involve selecting materials with low resistivity to ensure accurate sensing of pressure-induced electrical changes.
- Mechanical flexibility and stretchability: Conductive materials should possess mechanical properties that enable them to deform and stretch along with the sensor without compromising electrical conductivity. Materials with high flexibility and stretchability allow the sensor to conform to curved surfaces and undergo mechanical deformation without structural failure [83].
- Compatibility with substrates and integration: Conductive materials should be compatible with the chosen substrate and fabrication techniques for proper integration into the sensor structure [84]. Design strategies involve selecting materials that can be deposited, printed, or patterned onto the substrate using suitable techniques.
- Stability and reliability: Conductive materials should exhibit long-term stability and reliability under various environmental conditions, ensuring the sensor’s durability and consistent performance over time [85].
- Adhesion and interface compatibility: Conductive materials should have good adhesion properties to ensure strong bonding with the substrate and other components of the sensor, minimizing the risk of delamination or detachment during sensor operation [86].
- Scalability: Conductive materials should be readily available to enable the scalability and commercial viability of flexible and stretchable pressure sensors [87].
- Biocompatibility (for biomedical applications): In applications involving direct contact with the human body, conductive materials should be biocompatible to ensure compatibility with biological tissues and minimize the risk of adverse reactions [88].

### 3.2. Sensor Configurations

The sensor configurations in flexible and stretchable pressure sensors can vary depending on specific application requirements and the desired sensing mechanism. In this analysis, we will examine, explain, and review three common sensor configurations: thin film sensors, microstructured sensors, and textile-based sensors (Table 3).

#### 3.2.1. Thin Film Sensors

One common sensor configuration utilized in flexible and stretchable pressure sensors is thin film sensors. Thin films are typically composed of conductive materials with high electrical conductivity and mechanical flexibility. These films can be deposited onto flexible substrates, allowing the sensor to conform to curved surfaces and endure stretching and bending without compromising its performance. The choice of materials for thin film sensors plays a crucial role in determining their sensing capabilities. Conductive materials, such as carbon nanotubes, graphene, and conductive polymers, are commonly employed due to their excellent electrical properties. These materials enable efficient electrical signal transmission, resulting in high sensitivity and responsiveness to pressure changes. Thin film sensors offer several advantages in sensor configurations for flexible and stretchable pressure sensors. Firstly, their low profile and lightweight nature do not add significant bulk or weight to the overall sensor system, making them suitable for wearable and portable applications. Secondly, their mechanical flexibility allows them to endure mechanical deformations without structural failure, ensuring reliable and continuous pressure measurements. Furthermore, thin film sensors can be patterned into various shapes and sizes, enabling the design of sensor arrays for spatial pressure mapping. This capability enhances the sensor’s resolution and allows for more comprehensive pressure monitoring in complex applications. However, some challenges need to be addressed in the design and fabrication of thin film sensors. Ensuring good adhesion between the thin film and the substrate is essential to prevent delamination during stretching or bending.

#### 3.2.2. Microstructure Sensors

One notable sensor configuration is microstructure sensors. Microstructure sensors are characterized by the presence of carefully engineered microstructures on the sensor surface. These microstructures can be designed to enhance the sensor’s sensitivity, responsiveness, and mechanical properties. The design of microstructure sensors allows for improved pressure distribution and stress concentration, resulting in enhanced sensitivity to pressure variations. By tailoring the microstructure geometry and pattern, the sensor’s mechanical flexibility and stretchability can be optimized, ensuring reliable and accurate pressure measurements even under dynamic conditions. Various materials have been employed to create microstructure sensors, such as polymers, metals, and elastomers. Each material offers unique advantages in terms of mechanical properties and compatibility with specific applications. For instance, elastomers are often preferred due to their inherent stretchability and deformability, while metals provide excellent electrical conductivity for signal transmission. Microstructure sensors can be fabricated using various techniques, including microfabrication processes such as photolithography, laser ablation, and soft lithography. These techniques allow precise control over the microstructure design and enable the integration of multiple sensing elements into an array for enhanced spatial pressure mapping. One of the key challenges in designing microstructure sensors lies in achieving a balance between mechanical flexibility and structural integrity. The microstructures should withstand mechanical deformations without compromising the sensor’s functionality or leading to fatigue failure over time. Additionally, the sensor’s response to environmental factors, such as temperature and humidity changes, should be considered for stable and reliable long-term performance.

#### 3.2.3. Textile-Based Sensors

In recent years, there has been substantial growth and advancement in the field of textile-based pressure sensors. The trend is driven by the increasing demand for wearable electronics, health-monitoring devices, soft robotics, and human–machine interfaces that require sensors capable of conforming to irregular surfaces while maintaining comfort and flexibility for the users. Textile-based sensors have emerged as a promising solution to meet these requirements, leading to several noteworthy trends in this area:

- Integration of nanomaterials: Nanomaterial plays a vital role in the design and fabrication of textile-based sensors. It offers unique properties, such as high surface area and mechanical flexibility, which enhance the functionality of the sensors. Researchers have been exploring various nanomaterials, such as carbon nanotubes, graphene, metallic nanoparticles, 2D materials, and quantum dots incorporated with polymers to impart conductivity and sensing capabilities to textile substrates. The integration of nanomaterials into textiles allows for the creation of highly sensitive, stretchable, and conformable pressure sensors, enabling applications in areas such as smart clothing, medical devices, and sports monitoring.
- Hybrid sensor configurations: A notable trend in textile-based sensors is the emergence of hybrid sensor configurations that combine multiple sensing principles in a single device. For instance, some sensors incorporate both resistive and capacitive sensing elements to provide redundant measurements or improve sensitivity over a broader pressure range. The hybrid approach enables more versatile and multifunctional sensor designs, expanding their potential applications.
- Wearable and flexible electronics: With the increasing popularity of wearable electronics and smart textiles, textile-based pressure sensors have become an integral part of these devices. These sensors can be seamlessly integrated into garments, gloves, socks, and other wearable accessories to monitor body movements, health parameters, and environmental interactions. The trend of textile-based sensors has facilitated the development of wearable electronics with improved comfort and unobtrusiveness.
- Focus on signal processing and data analysis: As the complexity and volume of data generated by textile-based pressure sensors increase, there is a growing focus on signal processing and data analysis techniques. Researchers are exploring innovative algorithms and machine learning approaches to extract meaningful information from sensor data, enabling more accurate and real-time pressure monitoring. This trend enhances the practical utility of textile-based sensors in diverse applications.
- Application diversification: The trend of textile-based sensors is not limited to any specific industry or domain. Instead, these sensors find applications in a wide range of fields, including healthcare, sports and fitness, virtual reality, automotive, and industrial automation. As the technology matures, new applications continue to emerge, demonstrating the versatility and potential of textile-based pressure sensors.

### 3.3. Integration and Packaging Techniques

Integration and packaging techniques play a crucial role in ensuring the successful implementation and long-term performance of flexible and stretchable pressure sensors. This study aims to analyze, explain, and review three significant aspects of integration and packaging: stretchable electronics, soft substrate integration, and encapsulation and protection.

#### 3.3.1. Stretchable Electronics

Stretchable electronics involve the integration of electronic components, such as sensors, interconnects, and circuitry, onto stretchable substrates or materials. These components are designed to withstand mechanical deformation and strain without compromising their functionality. Various techniques, such as serpentine structures, meandering patterns, or island–bridge designs, enable flexibility and stretchability for distributing mechanical stress and strain across the electronics to minimize the risk of failure. Stretchable electronics allow for the seamless integration of sensors and electronics onto flexible and stretchable substrates, facilitating conformal contact with the target surface. They ensure reliable electrical connections and signal transmission even during mechanical deformation. However, challenges in stretchable electronics include ensuring electrical continuity under extreme strain, maintaining adhesion between different components, and managing the mechanical reliability of the integrated system.

#### 3.3.2. Soft Substrate Integration

Soft substrates refer to compliant and flexible materials that provide a deformable platform for integrating sensors and electronic components. These substrates can be elastomers, polymers, or flexible films capable of conforming to irregular shapes and withstanding mechanical deformations. Soft substrate integration involves methods such as direct deposition, transfer printing, or lamination to attach sensors and electronics onto the soft substrate. These techniques ensure good adhesion and compatibility between the different materials. Soft substrate integration enables conformal and comfortable contact with the target surface, such as the human body. It allows for natural motion and reduces the risk of discomfort or skin irritation. Additionally, soft substrates provide mechanical protection to the integrated components. However, challenges in soft substrate integration include achieving reliable bonding between different materials, managing the mechanical stress and strain on the interface, and optimizing the mechanical and electrical properties of the integrated system.

#### 3.3.3. Encapsulation and Protection

Encapsulation involves providing a protective layer or barrier around the integrated sensors and electronics to shield them from environmental factors, such as moisture, dust, and mechanical damage. It plays a crucial role in ensuring long-term stability, reliability, and durability. Encapsulation techniques can include conformal coating, encapsulation with elastomers or polymers, or encapsulation in flexible and transparent films. These techniques offer physical protection and environmental isolation with maintaining mechanical flexibility. Encapsulation and protection techniques safeguard the sensitive components from moisture, chemical exposure, and mechanical stress, enhancing the lifespan and performance of the sensor. They also aid in maintaining electrical insulation and preventing short circuits. However, challenges in encapsulation and protection include selecting materials with suitable properties, such as flexibility, transparency, and resistance to environmental factors. Additionally, achieving a conformal and uniform encapsulation layer without affecting sensor performance is essential.

## 4. Fabrication Techniques for Flexible and Stretchable Pressure Sensors

### 4.1. Printing and Deposition Methods

Printing and deposition methods for flexible and stretchable pressure sensors include screen printing, inkjet printing, aerosol jet printing, magnetron sputtering, vacuum evaporation, spin coating, spraying, and immersion/dip-coating techniques. These printing and deposition methods will be analyzed and explained in more detail.

#### 4.1.1. Screen Printing

Screen printing is a widely used fabrication technique in the production of flexible and stretchable pressure sensors. It involves the deposition of conductive or sensing materials through a screen or mesh onto a flexible substrate. The screen contains a stencil with the desired pattern, allowing for precise placement of the material on the substrate. This technique is cost-effective, scalable, and suitable for large-area production. The process of screen printing begins with preparing the stencil, which is usually made of a fine mesh material such as polyester or silk. The stencil is then attached to a frame, creating the screen. Next, the conductive or sensing material, such as conductive inks or polymers, is placed on the screen. A squeegee is used to force the material through the open areas of the stencil and onto the substrate. The thickness of the deposited material can be controlled by adjusting the pressure and speed of the squeegee. In the context of flexible and stretchable pressure sensors, screen printing can be used to deposit conductive tracks, electrodes, or sensing elements onto a variety of substrates, such as elastomers or flexible films. The choice of material and ink composition depends on the specific sensor requirements, including sensitivity, stretchability, and mechanical properties. One of the significant advantages of screen printing is its ability to achieve high-resolution patterns, which is crucial for creating precise sensor layouts. The technique is compatible with various conductive materials, allowing for the integration of multiple sensing elements onto the same substrate, enabling the creation of sensor arrays for pressure mapping [94]. However, screen printing still has some limitations. As it involves a sequential deposition process, it may not be suitable for applications requiring complex multilayer structures or intricate designs. Additionally, the printing resolution can be influenced by the mesh size, ink viscosity, and substrate properties, affecting the sensor’s overall performance.

#### 4.1.2. Inkjet Printing

Inkjet printing is a versatile and innovative fabrication technique utilized in the production of flexible and stretchable pressure sensors. It involves the precise deposition of functional materials, such as conductive inks or sensing polymers, in a controlled and digitally programmable manner. Inkjet printing offers several advantages, including high resolution, scalability, and the ability to create complex patterns. The inkjet printing process starts with the formulation of the conductive or sensing ink, which must possess suitable rheological properties to ensure stable and controlled droplet formation during printing. The ink is loaded into ink cartridges, and the printer is programmed to dispense the ink droplets onto the flexible substrate according to the desired sensor design. Inkjet printing offers excellent resolution and control over the droplet size and placement, enabling the creation of intricate patterns with high precision. This capability is particularly advantageous for fabricating flexible and stretchable pressure sensors, where fine and accurate sensing elements are essential for achieving reliable pressure measurements. One of the significant benefits of inkjet printing is its non-contact nature, which minimizes mechanical stress on the substrate and the sensing elements. This non-contact printing allows for the deposition of materials onto delicate and stretchable substrates without causing damage or deformation, preserving the sensor’s mechanical flexibility. Furthermore, inkjet printing allows for the digital and programmable nature of the fabrication process. This feature enables rapid prototyping and customization of sensor designs, facilitating the development of tailored sensors for specific applications. It also supports the creation of sensor arrays or complex multilayer structures, expanding the potential applications of flexible and stretchable pressure sensors [89]. However, there are some challenges associated with inkjet printing for sensor fabrication. The choice of ink and substrate materials is critical to ensure good adhesion and compatibility between the layers. Additionally, optimizing the ink formulation and printer settings is crucial to achieving consistent droplet properties and uniformity across the printed patterns.

#### 4.1.3. Aerosol Jet Printing

Aerosol jet printing is an advanced additive-manufacturing technique used in the fabrication of flexible and stretchable pressure sensors. This method involves the deposition of functional materials, such as conductive inks or sensing polymers, using a focused aerosol stream. Aerosol jet printing offers several advantages, including high resolution, precision, and the ability to print on curved and non-planar surfaces. The aerosol jet printing process starts with the formulation of the functional ink into aerosol droplets. The ink is then loaded into a nebulizer, which converts it into a fine aerosol mist. The aerosol is directed through a nozzle, and a gas sheath surrounds it to focus the aerosol stream into a narrow and well-controlled jet. This focused aerosol stream is then precisely directed onto the flexible substrate, enabling the deposition of the functional material. One of the significant advantages of aerosol jet printing is its high resolution and precise control over the deposition process. The fine aerosol jet allows for printing fine patterns and intricate designs with excellent accuracy, enabling the creation of detailed sensing elements in pressure sensors [90]. This capability is particularly beneficial for applications where high-resolution pressure mapping is required. Another advantage of aerosol jet printing is its ability to print on curved and non-planar surfaces. The focused aerosol stream can adapt to the contour of the substrate, enabling conformal printing of sensing elements onto irregular shapes. This feature is essential for fabricating pressure sensors that can conform to the surface of the target object or body, ensuring accurate pressure measurements. Moreover, aerosol jet printing is a non-contact and additive process, which minimizes mechanical stress on the substrate and sensing elements. This non-contact nature allows for the deposition of materials on delicate and stretchable substrates without causing damage or deformation, preserving the sensor’s mechanical flexibility. However, there are some challenges associated with aerosol jet printing for sensor fabrication. The choice of ink formulation and nozzle design is critical to achieving consistent droplet properties and uniformity in the printed patterns. Additionally, optimization of the printing parameters and control over the aerosol jet velocity are essential for maintaining the precision and resolution of the printed features.

#### 4.1.4. Magnetron Sputtering

Magnetron sputtering is a physical vapor deposition technique. It involves the deposition of thin films of functional materials, such as conductive metals or semiconductors, onto a flexible substrate. Magnetron sputtering offers several advantages, including precise control over film thickness, high deposition rates, and excellent material adhesion. In the magnetron sputtering process, a target material (the source of the thin film) is placed in a vacuum chamber. The flexible substrate is also positioned within the chamber, facing the target material. When a high-energy plasma is generated within the chamber, positively charged ions from the plasma bombard the target material. This causes the ejection of atoms from the target surface, which then condense onto the substrate, forming a thin film. One of the significant advantages of magnetron sputtering is its ability to precisely control the film thickness and composition. By adjusting the deposition parameters, such as the gas pressure, power, and target–substrate distance, the film’s thickness can be tailored to meet the specific requirements of the pressure sensor. This control over film thickness is crucial for achieving the desired electrical and mechanical properties of the sensor. Magnetron sputtering also allows for high deposition rates, enabling the rapid and efficient production of flexible and stretchable pressure sensors [95]. This feature is advantageous for large-scale manufacturing where high throughput is required. Moreover, magnetron sputtering provides excellent adhesion between the thin film and the flexible substrate. The sputtering process promotes strong bonding between the deposited material and the substrate, ensuring the mechanical integrity of the sensor even under strain or deformation. However, there are some challenges associated with magnetron sputtering for sensor fabrication. The high-energy plasma used in the process can damage the flexible substrate or alter its mechanical properties. Careful selection of the process conditions and substrate materials is necessary to mitigate these issues. Additionally, the sputtering equipment can be complex and expensive, requiring specialized knowledge for operation and maintenance.

#### 4.1.5. Vacuum Evaporation

Vacuum evaporation is a common thin film deposition technique. It involves the vaporization of a material in a vacuum environment, followed by the condensation of the vapor onto a flexible substrate. Vacuum evaporation offers several advantages, including precise control over film thickness, uniformity, and the ability to deposit a wide range of materials. In the vacuum evaporation process, the material to be deposited (known as the evaporant) is placed in a crucible or boat inside a vacuum chamber. The chamber is then evacuated to create a low-pressure environment, which prevents unwanted reactions and ensures a controlled deposition process. When the material is heated, it undergoes sublimation and transforms into a vapor. The vaporized material then condenses onto the substrate, forming a thin film. One of the significant advantages of vacuum evaporation is its ability to precisely control the film thickness. By monitoring the deposition time and evaporation rate, the desired film thickness can be achieved, ensuring the sensor’s optimal performance. Vacuum evaporation also allows for uniform deposition across the entire substrate surface [96]. This feature is crucial for producing consistent and reliable pressure sensors with uniform sensing elements. Moreover, vacuum evaporation is compatible with a wide range of materials, including metals, semiconductors, and insulators. This versatility enables the fabrication of various types of pressure sensors with different electrical and mechanical properties. However, there are some challenges associated with vacuum evaporation for sensor fabrication. Some materials may require higher evaporation temperatures, which can be problematic for flexible and stretchable substrates that are sensitive to heat. Furthermore, vacuum evaporation is typically a line-of-sight deposition technique, which may result in poor coverage of three-dimensional and non-planar substrates. Additional steps, such as rotation or tilt of the substrate, may be required to achieve conformal coatings on irregular surfaces.

#### 4.1.6. Spin Coating

Spin coating is a widely used deposition technique in the fabrication of flexible and stretchable pressure sensors. It involves the application of a liquid material, such as a solution or suspension of functional materials, onto a rotating substrate. As the substrate spins, the centrifugal force spreads the liquid material uniformly, creating a thin and homogeneous film. Spin coating offers several advantages, including simplicity, uniformity, and the ability to deposit a wide range of materials. In the spin-coating process, the liquid material is dispensed onto the center of the substrate. The substrate is then rapidly spun, causing the liquid to spread out radially and form a thin film. The spinning speed and duration are carefully controlled to achieve the desired film thickness and uniformity [97]. One of the significant advantages of spin coating is its simplicity and ease of implementation. The process can be performed with relatively simple equipment and does not require complex deposition systems. This makes spin coating a cost-effective and accessible technique for producing pressure sensors. Spin coating also enables the deposition of uniform and smooth films over large areas. The spinning action ensures a consistent distribution of the liquid material, resulting in films with minimal thickness variation. This uniformity is essential for pressure sensors to ensure consistent and accurate measurements. Moreover, spin coating is compatible with a wide range of materials, including conductive materials, semiconductors, and polymers. This versatility allows for the deposition of different types of sensing elements or conductive tracks, tailored to the specific requirements of the pressure sensor. However, there are some challenges associated with spin coating for sensor fabrication. The film thickness achieved by spin coating can be sensitive to factors such as the viscosity and concentration of the liquid material, the spinning speed, and the ambient conditions. Careful optimization of these parameters is necessary to achieve the desired film properties. Furthermore, spin coating is generally a planar deposition technique which may result in limited coverage on three-dimensional or non-planar substrates. Additional steps, such as multiple spin coating or other deposition techniques, may be required to achieve conformal coatings on irregular surfaces.

#### 4.1.7. Spraying

Aerosol spraying or spray-coating techniques involve the deposition of functional materials onto a flexible substrate by spraying a liquid solution or suspension containing the materials. Spraying offers several advantages, including simplicity, scalability, and the ability to coat large areas. In aerosol spraying or spray coating, the functional material is dissolved or suspended in a liquid solvent to form a sprayable solution. The solution is then loaded into a spray gun or nozzle, which atomizes the liquid into fine droplets. These droplets are then directed onto the flexible substrate using a carrier gas or compressed air. As the droplets impact the substrate, they coalesce and form a thin film [98]. One of the significant advantages of spraying techniques is their simplicity and ease of implementation. The process can be carried out with relatively simple equipment, making it a cost-effective and accessible option for producing pressure sensors. Spraying also allows for the deposition of films over large areas, making it suitable for coating large substrates or producing sensor arrays. This scalability is advantageous for high-throughput manufacturing and large-scale sensor production. Moreover, spraying techniques are compatible with various materials, including conductive inks, polymers, and nanoparticles, providing flexibility in designing the sensing elements or conductive tracks for the pressure sensor. However, there are some challenges associated with spraying techniques for sensor fabrication. The film thickness achieved by spraying can be sensitive to factors such as the concentration of the functional material in the solution, the spraying distance, and the nozzle size. Careful optimization of these parameters is necessary to control the film thickness and ensure consistent performance of the pressure sensor. Additionally, spraying may result in some non-uniformity in the film thickness, particularly at the edges of the sprayed area. This non-uniformity may require post-processing steps, such as multiple spraying or manual touch-ups, to achieve a more uniform coating.

#### 4.1.8. Immersion/Dip Coating

Immersion or dip coating involves immersing a flexible substrate into a solution containing the functional materials, followed by withdrawing the substrate at a controlled speed. As the substrate is withdrawn, a thin film of the solution adheres to the surface, forming a coating. Immersion coating offers several advantages, including simplicity, controllable film thickness, and the ability to coat complex and irregular substrates. In the immersion-coating process, the functional material is dissolved or suspended in a liquid solvent to form a coating solution. The flexible substrate is then dipped into the solution, allowing the solution to coat the entire substrate surface [37]. The withdrawal speed and the duration of immersion are carefully controlled to achieve the desired film thickness. One of the significant advantages of immersion coating is its simplicity and ease of implementation. The process requires minimal equipment, making it a cost-effective and accessible option for producing pressure sensors. Immersion coating also allows for precise control over the film thickness. By adjusting the immersion time and withdrawal speed, the film’s thickness can be tailored to meet the specific requirements of the pressure sensor. This control is crucial for achieving the desired electrical and mechanical properties of the sensor. Moreover, immersion coating is suitable for coating complex and irregular substrates. The immersion process ensures that the coating solution adheres uniformly to the substrate, even on non-planar surfaces. This feature is beneficial for fabricating pressure sensors that need to conform to irregular shapes or surfaces [99]. However, there are some challenges associated with immersion coating for sensor fabrication. The uniformity of the coating can be influenced by factors such as the viscosity of the solution, the withdrawal speed, and the temperature of the solution. Careful optimization of these parameters is necessary to achieve a uniform and consistent coating. Additionally, immersion coating may result in some solvent retention in the film, which can affect the sensor’s electrical and mechanical properties. Proper drying or post-processing steps may be required to remove any residual solvent and improve the film’s performance.

### 4.2. Microfabrication and Patterning

Microfabrication and patterning techniques play a pivotal role in advancing the development of flexible and stretchable pressure sensors. In this context, three techniques, including photolithography, soft lithography, and laser machining will be discussed in the following subsections.

#### 4.2.1. Photolithography

Photolithography uses light to transfer a pattern onto a photosensitive material, typically a photoresist, on a substrate. Photolithography enables the creation of precise and well-defined microstructures, such as electrodes, sensing elements, or conductive tracks, which are essential components of pressure sensors. The photolithography process starts with the application of a photosensitive material onto the substrate. The photoresist is a light-sensitive polymer that undergoes a chemical change upon exposure to UV light. The photoresist-coated substrate is then placed under a photomask, which contains the desired pattern in opaque and transparent regions. When exposed to UV light, the photoresist in the transparent regions of the photomask becomes soluble and is removed during the development step, leaving behind the desired pattern on the substrate [100]. The photoresist in the opaque regions remains insoluble and acts as a protective mask during subsequent processing steps. The developed pattern serves as a template for subsequent material deposition or etching steps. For example, in the context of flexible and stretchable pressure sensors, the patterned photoresist can be used as a mask for depositing conductive materials, such as metals or conductive polymers, onto the substrate. Alternatively, it can be used as a mask for etching away unwanted material to create microstructures. One of the significant advantages of photolithography is its ability to achieve high-resolution patterning. The photomasks can be created with extremely fine features, allowing for the fabrication of precise and intricate microstructures. This level of precision is crucial for producing high-performance pressure sensors with well-defined sensing elements. Photolithography is also a scalable and repeatable process, making it suitable for the mass production of pressure sensors. The photomasks can be replicated and reused, enabling the production of identical patterns on multiple substrates. However, photolithography requires a clean and controlled environment to avoid contamination of the photoresist.

#### 4.2.2. Soft Lithography

Soft lithography enables the transfer of patterns from a mold to a soft elastomeric material that can be used to create well-defined microstructures on the substrate. Soft lithography offers several advantages, including simplicity, high resolution, and compatibility with a variety of substrates. The soft lithography process begins with the creation of a master mold, typically made from silicon or other materials. The mold is fabricated by using traditional lithography and etching techniques or advanced micro/nanofabrication processes. Once the master mold is ready, it is coated with a thin layer of a liquid elastomer, commonly polydimethylsiloxane (PDMS), to create a replica mold. The PDMS replica mold is then carefully peeled off from the master mold, carrying with it the patterned features. Next, the PDMS replica mold is placed on the target substrate and pressure/a gentle conformal contact is applied to transfer the pattern onto the substrate. The elastomeric nature of PDMS allows it to conform to the substrate’s surface, creating a conformal and precise microstructure pattern [101]. Soft lithography enables the fabrication of microstructures with high resolution and aspect ratios, which are essential for creating intricate sensing elements and conductive tracks in pressure sensors. The technique is well-suited for creating flexible and stretchable patterns, allowing the pressure sensors to conform to curved or irregular surfaces without compromising their performance. Moreover, soft lithography is a gentle process that can be performed at room temperature, minimizing the risk of damaging delicate and flexible substrates. It also offers scalability, allowing the creation of arrays of sensors or large-scale production. However, there are some challenges associated with soft lithography. The replication fidelity of the mold pattern onto the substrate can be influenced by factors such as the quality of the master mold, the choice of elastomeric material, and the application of pressure during the transfer process. Careful control of these parameters is necessary to ensure accurate pattern transfer and consistency in the sensor’s performance.

#### 4.2.3. Laser Machining

Laser machining is a precise and versatile microfabrication technique utilized in the step of patterning for flexible and stretchable pressure sensor fabrication. It involves the use of a laser beam to selectively remove material from the substrate, creating well-defined microstructures and conductive patterns [91]. Laser machining offers several advantages, including high precision, non-contact processing, and the ability to work with a wide range of materials. The laser-machining process begins with the design of the desired microstructure or pattern using computer-aided design (CAD) software. The laser parameters, such as intensity, pulse duration, and scanning speed, are carefully set based on the material and desired features to be created. The laser beam is then focused onto the substrate, and the material at the focal point absorbs the laser energy, leading to localized heating and vaporization. This process allows for the precise removal of material, leaving behind the pattern or microstructure on the substrate. Laser machining offers high resolution and precise control over the pattern’s dimensions, allowing for the fabrication of intricate and complex microstructures that are crucial for pressure sensors [102]. The non-contact nature of laser machining minimizes mechanical stress on the substrate, making it suitable for delicate and flexible materials commonly used in flexible sensors. The versatility of laser machining enables it to work with various materials, including polymers, metals, and ceramics, providing flexibility in designing the sensing elements or conductive tracks for pressure sensors. Moreover, laser machining is a fast and efficient process, making it suitable for both prototyping and the large-scale production of pressure sensors. Its digital nature also allows for rapid design iterations and adjustments, streamlining the development process. However, there are some challenges associated with laser machining. The selection of laser parameters and the choice of suitable materials are critical to achieving the desired pattern with minimal heat-affected zones or material damage. Laser machining may also require additional post-processing steps to clean the debris generated during the process.

### 4.3. Assembly and Integration Processes

#### 4.3.1. Transfer Printing

Transfer printing involves the transfer of microscale or nanoscale structures such as sensing elements or conductive tracks from their original substrate onto a flexible and stretchable platform. Transfer printing offers several advantages, including high precision, compatibility with various materials, and the ability to create complex sensor configurations. The transfer-printing process begins with the fabrication of the microstructures or sensing elements on a sacrificial substrate, often made from rigid materials such as silicon or glass. These microstructures can be created using various microfabrication techniques, including photolithography, soft lithography, or laser machining [103]. Once the microstructures are fabricated. A transfer layer, usually made from a thin elastomeric material like PDMS, is coated on top of the sacrificial substrate. The transfer layer acts as a stamp adhering to the microstructures upon contact. Next, the transfer layer with the attached microstructures is brought into conformal contact with the target flexible and stretchable substrate. Pressure or gentle mechanical forces are applied to ensure good adhesion and transfer of the microstructures. Once the transfer process is complete, the transfer layer is carefully peeled off, leaving the microstructures firmly attached to the flexible substrate. The sacrificial substrate can be dissolved or removed using appropriate etching techniques, leaving behind the transferred microstructures on the flexible and stretchable platform. Transfer printing allows for the precise integration of microstructures onto the flexible and stretchable substrate, enabling the creation of intricate and well-defined sensing elements or conductive tracks for pressure sensors. The process is versatile and compatible with a wide range of materials, making it suitable for different sensor configurations and material combinations. The high precision and alignment capabilities of transfer printing allow for the creation of complex sensor arrays or multi-layered sensor structures, enhancing the sensor’s performance and capabilities. Moreover, transfer printing is a gentle and non-destructive process, minimizing the risk of damaging the delicate flexible substrates commonly used in pressure sensors. However, there are some challenges associated with transfer printing. Achieving the uniform and consistent transfer of microstructures across large areas may be challenging and may require careful optimization of the process parameters. Additionally, the adhesion and compatibility between the transfer layer and the microstructures, as well as the target flexible substrate, need to be carefully managed to ensure successful transfer printing.

#### 4.3.2. Direct Integration Techniques

Direct integration involves directly integrating the sensing elements or conductive tracks onto the flexible and stretchable substrate without the need for additional transfer or assembly steps [92]. There are several direct integration techniques commonly employed in pressure sensor fabrication, each offering unique advantages and considerations. One such technique is direct printing, where conductive materials, such as conductive inks or pastes, are directly deposited onto the flexible substrate using methods such as screen printing, inkjet printing, or aerosol jet printing. These printing techniques allow for the precise deposition of conductive patterns and sensing elements, enabling the creation of flexible and stretchable pressure sensors with specific designs and configurations. The advantages of direct printing include high precision and resolution in pattern deposition, a fast and cost-effective process suitable for large-scale production, and compatibility with various materials and substrates [93]. However, careful consideration is needed in the choice of ink or paste and substrate compatibility to ensure the sensor’s performance and durability. Additionally, the optimization of printing parameters is necessary to achieve uniform and consistent patterns. Another direct integration technique is 3D printing, also known as additive manufacturing, which is used to create three-dimensional pressure sensors directly on the flexible substrate. This technique involves a layer-by-layer deposition of materials to build complex and customized sensor structures. Three-dimensional printing allows for the creation of intricate and custom-designed sensor architectures, enabling the integration of multiple sensing elements and functionalities in a single structure. However, it has limitations in resolution and accuracy compared to traditional microfabrication techniques. The material selection and printing parameters can significantly influence the mechanical and electrical properties of the final sensor.

Advantages and limitations of fabrication techniques for flexible and stretchable pressure sensors are summarized in Table 4.

## 5. State-of-the-Art Applications of Flexible and Stretchable Pressure Sensors

### 5.1. Healthcare and Biomedical Applications

**Figure 5:**
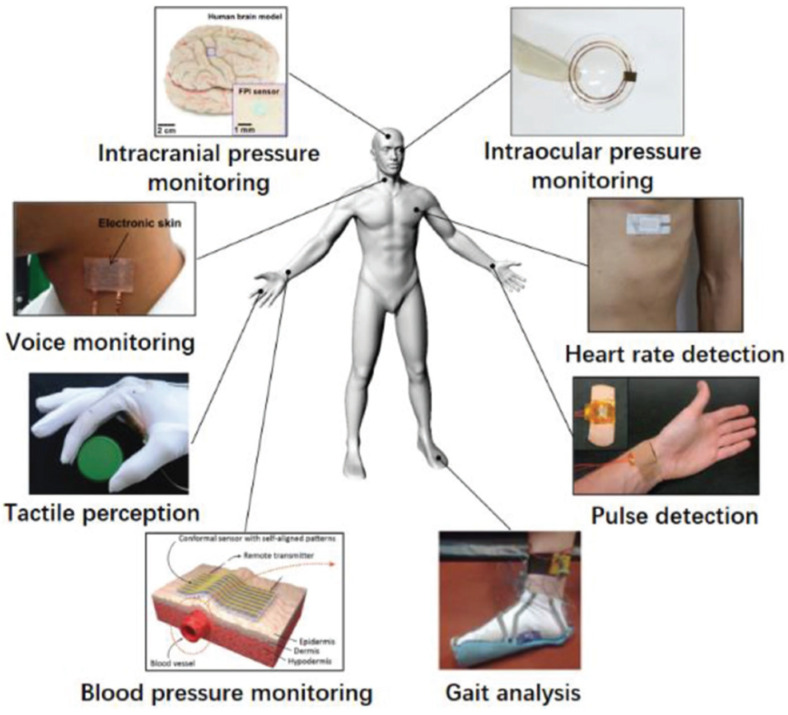
Healthcare and biomedical applications of flexible and stretchable pressure sensors. (Reproduced with permission from John Wiley & Sons [106]).

Flexible and stretchable pressure sensors have emerged as promising technologies in healthcare and biomedical applications, as shown in Figure 5. Their unique properties, such as conformability, stretchability, and sensitivity, make them ideal for monitoring physiological parameters and detecting health-related conditions. One key application is continuous physiological monitoring where these sensors are integrated into wearable devices or smart textiles to monitor vital signs, such as blood pressure, heart rate, respiratory rate [90], and body motion [104]. Wearable wristbands with embedded pressure sensors enable non-invasive blood pressure monitoring, facilitating real-time cardiovascular health tracking. Conformable sensors placed on the chest accurately measure respiratory rate and detect abnormal breathing patterns, aiding in respiratory disorder diagnosis and management. Moreover, flexible and stretchable pressure sensors find applications in wound monitoring and pressure ulcer prevention. Integrated into bandages or dressings, they provide continuous pressure distribution monitoring at the wound site, ensuring optimal healing conditions and preventing excessive pressure on vulnerable areas. Real-time feedback enables the early detection of compromised blood flow and pressure-induced tissue damage, reducing the risk of pressure ulcers and improving patient care. The integration of these sensors into prosthetic limbs and rehabilitation devices shows potential for enhancing functionality and patient outcomes [105]. Detecting pressure distribution and force exertion allows for better control and natural interaction with prosthetic limbs, leading to more precise and intuitive movements and improved mobility. In robotic-assisted surgical systems, flexible and stretchable pressure sensors enhance safety, precision, and tactile feedback during procedures [98]. Surgeons gain real-time information on applied forces, tissue compliance, and contact pressure, enabling more accurate tissue manipulation and reducing the risk of tissue damage. Furthermore, incorporating these sensors into drug delivery systems enables personalized and controlled medication administration. Real-time monitoring of pressure changes in fluidic channels or implanted devices ensures accurate drug infusion rates and enhances patient safety. The sensors also provide valuable feedback on drug diffusion and tissue response, optimizing treatment protocols and improving therapeutic outcomes.

### 5.2. Human–Machine Interfaces and Robotics

**Figure 6:**
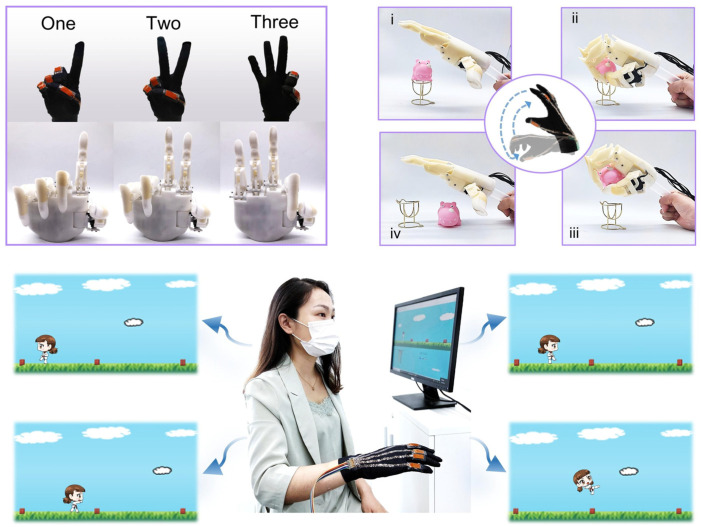
Human–machine interface applications of flexible and stretchable pressure sensors. Note that (**i**–**iv**) step process for grab object. (Reproduced with permission from Elsevier [107]).

Flexible and stretchable pressure sensors have garnered significant attention in the field of human–machine interfaces (HMIs), as shown in Figure 6, due to their ability to detect and respond to touch, pressure, and force [107]. By integrating these sensors into touchscreens and wearable devices, users can interact with machines and digital interfaces through touch and gestures [108]. For example, wearable devices with embedded pressure sensors can detect hand gestures and movements, enabling intuitive control of electronic devices, appliances, and gaming interfaces [109]. These sensors play a vital role in robotics and prosthetics, facilitating the development of more advanced and responsive robotic systems and prosthetic limbs. Embedded within robotic hands [110], grippers [111], and prosthetic limbs, they provide tactile sensing capabilities, enabling robots and amputees to perceive and respond to touch and pressure [112]. By detecting and measuring forces exerted on surfaces or objects, these sensors enhance the grasping and manipulation abilities of robots and provide amputees with enhanced proprioception, allowing for more natural and intuitive control of prosthetic limbs. In the field of virtual reality (VR) interfaces [113], flexible and stretchable pressure sensors contribute to more immersive and interactive experiences. Integrated into gloves [114], bodysuits, or haptic devices, they capture and transmit the user’s touch and pressure feedback in virtual environments. By accurately detecting hand movements, finger motions, and tactile sensations, these sensors enhance the realism and interactivity of VR simulations, providing users with a more immersive and engaging experience [115]. They also enable haptic feedback, allowing users to feel virtual objects and textures, enhancing the sense of presence in virtual environments.

**Figure 7:**
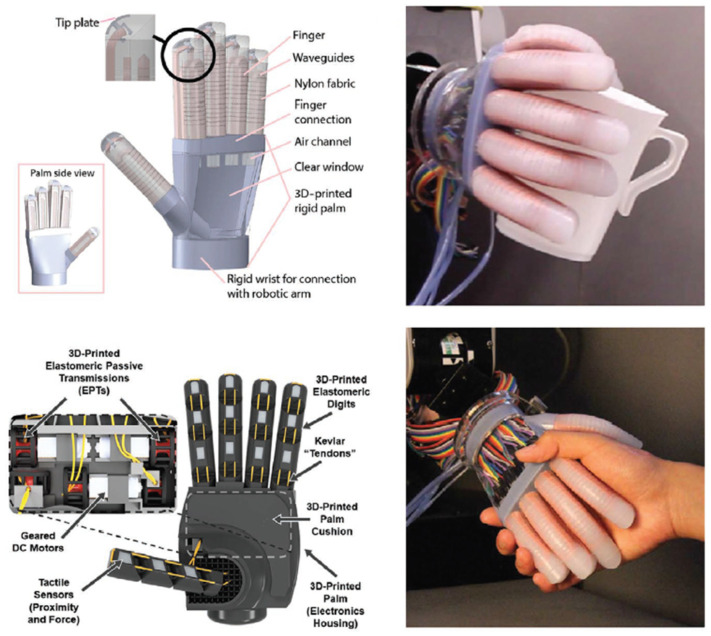
Robotics application of flexible pressure (force) sensors. (Reproduced with permission from John Wiley & Sons [118]).

Flexible and stretchable pressure sensors provide robots with tactile sensing capabilities, enabling them to interact with the physical environment, as shown in Figure 7. Integrated into robot fingertips [116], grippers, and skin [117], they can detect contact forces, pressure distribution, and object properties, allowing for adjustments in grip force and safe interactions with humans. Integration into robot arms and legs enables force control, resulting in more precise movements and safer interactions with the environment. In prosthetics, these sensors play a crucial role in providing real-time pressure and force feedback to prosthetic hands, fingers, and feet [118]. Amputees can adjust grip strength to detect object slippage and perform delicate tasks with improved dexterity. Integrated into the prosthetic socket, these sensors offer valuable information about pressure distribution, optimizing socket design and customization, enhancing comfort, and reducing the risk of pressure sores. In robotic-assisted rehabilitation, these sensors are integrated into exoskeletons and assistive devices to monitor pressure during movement and exercises, providing real-time feedback for the precise control of robotic assistance. This technology enables personalized and adaptive rehabilitation programs, optimizing recovery and motor skill development.

### 5.3. Internet of Things (IoT) and Smart Systems

Flexible and stretchable pressure sensors can demonstrate their potential impact on creating intelligent environments and optimizing resource allocation. In structural health monitoring, these sensors play a crucial role in ensuring infrastructure, building, and machinery integrity and safety. Embedded in materials such as concrete or composites, they monitor pressure and strain, detecting structural weaknesses, damage, or excessive loads. Real-time monitoring enables proactive maintenance, reducing failure risk and extending infrastructure lifespan. In smart home and building automation, flexible and stretchable pressure sensors create intelligent environments that adapt to human needs and preferences [119]. Integrated into furniture, shoes [120], walls, floors, or HVAC systems, they monitor occupancy, optimize energy consumption, and enhance user comfort. For example, sensors in seats or beds detect occupancy and adjust lighting and HVAC settings, accordingly, promoting energy efficiency and personalized control. Automotive applications benefit from these sensors, enhancing safety, comfort, and performance. Integrated into automotive interiors, such as seats, steering wheels, and pedals, they monitor driver and passenger posture, occupancy, and pressure distribution [121]. The data analysis enables airbag deployment adjustments and optimal seating positions. Pressure sensors also monitor tire pressure, improving vehicle control for safety and fuel efficiency [122]. In industrial settings, flexible and stretchable pressure sensors enable the real-time monitoring of machinery, equipment, and manufacturing processes. Integrated into industrial equipment, they detect pressure changes, optimize resource allocation, and ensure operational efficiency. Pneumatic systems with embedded pressure sensors monitor pressure levels, adjusting airflow for energy conservation and productivity enhancement. Environmental-monitoring applications utilize these sensors to measure pressure and force in natural and built environments. Deployed in soil, water, or air, they monitor parameters such as water pressure in pipelines, soil compaction, or air pressure in HVAC systems [123]. Continuous monitoring optimizes environmental conditions and allows for prompt issue detection and resolution. The latest advances in the application of flexible and stretchable pressure sensors in smart farms and fish farming have brought about a revolution in farming practices, leading to enhanced productivity, resource efficiency, and sustainable agriculture [124]. These sensors play a crucial role in monitoring various parameters within the agricultural environment, empowering farmers to make data-driven decisions and optimize their operations. Among the key advancements, plant-pressure monitoring stands out as an important application. Pressure sensors placed on plants’ surfaces facilitate the real-time monitoring of plant health and stress levels [125]. These sensors measure turgor pressure, which provides insights into water content and hydration status within plant cells [126]. Analyzing this data enables farmers to identify signs of drought stress or disease promptly, enabling timely interventions and efficient water management strategies [127].

The type of materials, sensing mechanisms, and performance that are related to flexible and stretchable pressure sensors are summarized in Table 5.

## 6. Challenges and Future Directions

### 6.1. Performance Improvement

One of the primary challenges in flexible and stretchable pressure sensors is achieving high sensitivity, accuracy, and stability. Researchers are focusing on improving the sensor’s signal-to-noise ratio, linearity, and dynamic range. Based on recent reviews, numerous studies have reported the development of flexible pressure sensors exhibiting a wide linear dynamic detection range, with high thresholds starting from 200 kPa and exceeding this value [94,105,136,141]. Of particular note is the remarkable achievement of an unprecedented ultrahigh sensitivity, reaching a maximum sensitivity of approximately 173,688 kPa^−^^1^. This involves exploring new materials with enhanced mechanical and electrical properties, optimizing sensor designs and architectures, and developing advanced signal-processing techniques. Future directions include the integration of multiple sensing mechanisms and the use of nanomaterials and nanofabrication techniques to enhance the performance of these sensors.

### 6.2. Durability and Reliability

Flexible and stretchable pressure sensors face challenges related to long-term durability and reliability. Repeated mechanical deformation and environmental exposure can lead to material fatigue, degradation of electrical properties, and failure of sensor components. Future research efforts are aimed to develop robust and resilient materials, such as self-healing polymers or composite structures, that can withstand cyclic strains and maintain sensor functionality over extended periods. Presently, the majority of studies indicate long-term durability values utilized in testing to fall within the range of 10,000 to 20,000 cycles [153,154], accompanied by certain reports suggesting long-term durability extending up to 50,000 cycles [155]. Additionally, the development of encapsulation techniques and protective coatings can enhance the sensor’s resistance to moisture, chemicals, and mechanical stress, ensuring long-term reliability.

### 6.3. Biocompatibility and Safety

For applications involving direct contact with the human body, biocompatibility and safety are of importance. Flexible and stretchable pressure sensors must be designed and fabricated using materials that are non-toxic, hypoallergenic, and compatible with biological systems. Research is focused on exploring biocompatible materials, such as bioresorbable polymers or biocompatible coatings, that minimize the risk of adverse reactions or infections. Examples of such materials include bioresorbable poly(l-lactide) and glycine [156]. Future directions include in-depth biocompatibility testing, standardization of safety guidelines, and the development of sensor integration methods that ensure safe and reliable operation in healthcare and biomedical applications [157].

### 6.4. Emerging Trends and Future Applications

Flexible and stretchable pressure sensors hold tremendous potential for emerging trends and future applications. One such trend is the integration of multiple-sensing modalities, such as temperature, humidity, or strain, into a single sensor platform, enabling multifunctional sensing capabilities [158,159]. Additionally, the integration of artificial intelligence and machine learning algorithms can enable real-time data analysis, pattern recognition, and predictive modeling, leading to advanced monitoring and diagnostic capabilities [160,161]. Future applications include personalized healthcare [162], sports and fitness monitoring [163], virtual and augmented reality interfaces, human–robot collaboration, and intelligent wearable devices. Researchers are also exploring novel integration approaches, such as 3D printing or textile-based integration, to expand the possibilities and versatility of flexible and stretchable pressure sensors.

## 7. Summary

This comprehensive review examines the emergence of flexible and stretchable pressure sensors as a highly promising technology with vast potential across numerous applications. This review encompasses an in-depth analysis of the fundamental principles, design strategies, fabrication techniques, and state-of-the-art applications of these sensors. The study delves into the various sensing mechanisms employed, such as resistive, capacitive, and piezoelectric, highlighting their distinct advantages and limitations. It emphasizes the importance of selecting the most suitable mechanism based on specific application requirements. Design strategies are explored, with a particular focus on material selection, including elastomers and conductive materials, as they significantly impact the sensors’ mechanical flexibility, electrical conductivity, and biocompatibility. The discussed design strategies offer valuable insights for enhancing sensor performance and functionality. The review also investigates various fabrication techniques, including screen printing, inkjet printing, aerosol jet printing, vacuum evaporation, spin coating, spraying, and immersion/dip coating deposition. It examines their roles in manufacturing flexible and stretchable pressure sensors, along with their advantages and limitations. These fabrication techniques enable scalable and cost-effective production while ensuring mechanical flexibility and high performance. This study further explores different sensor configurations, such as thin film sensors, microstructure sensors, and textile-based sensors. It highlights their unique benefits, such as conformability, wearability, and integration with diverse substrates, enabling applications in healthcare, robotics, prosthetics, and beyond. Integration and packaging techniques, including stretchable electronics, soft substrate integration, and encapsulation methods, are discussed, emphasizing their significance in seamlessly integrating sensors into various systems. They ensure reliable operation under dynamic conditions and provide protection against environmental factors. This review also explores the state-of-the-art applications of flexible and stretchable pressure sensors across healthcare and biomedical applications, human–machine interfaces, robotics and prosthetics, and the Internet of Things (IoT) and smart systems. It demonstrates their potential to revolutionize these domains and foster innovation. However, despite significant progress, challenges remain, including the improvement of performance, durability, and reliability, ensuring biocompatibility and safety, and addressing emerging trends. Future research efforts should focus on overcoming these challenges, ultimately promoting the widespread adoption and integration of flexible and stretchable pressure sensors in various industries. This advancement will further advance wearable electronics, healthcare diagnostics, human–machine interactions, and other fields reliant on pressure monitoring.

## Figures and Tables

**Table 1:** Comparison of pressure-sensing mechanisms.

Sensing Mechanisms	Sensor Properties	Advantages	Disadvantages	Year [Reference]
Piezoresistive	Sensitivity	- High sensitivity to pressure changes. - Suitable for precise pressure measurements.	- Sensitivity affected by environmental factors. - May experience drift over time.	2022 [32] 2020 [33]
	Range of measurement	- Wide range of pressure handling capability, from low to high values.	- Limited measurement ranges; in certain cases, due to material constraints.	2020 [34]
	Mechanical durability	- Mechanically durable, especially in robust structures.	- Mechanical durability may be affected by excessive bending or stretching.	2020 [35]
	Fabrication complexity	- Relatively simple fabrication using common materials and straightforward techniques.	- Challenging integration into flexible or stretchable formats.	2019 [36]
	Power consumption	- Low power requirements, offering energy efficiency for specific applications.	- Power consumption may increase during active signal conditioning and readout processes.	2021 [37]
Capacitive	Sensitivity	- Good sensitivity, especially in detecting small pressure variations. - Less affected by temperature changes compared to resistive sensors.	- Sensitivity to external interference or noise, impacting accuracy.	2022 [38]
	Range of measurement	- Covers a broad range of pressure values. - Provides versatility in various applications.	- Dielectric properties may be affected by extreme pressure conditions. - Limited upper measurement range.	2019 [39]
	Mechanical durability	- Designed with flexible and stretchable materials. - Enhanced mechanical durability and resilience.	- Degradation in performance due to repeated stretching and flexing over time.	2022 [40]
	Fabrication complexity	- Fabricated using flexible and stretchable materials. - Enables diverse design possibilities.	- Manufacturing process may involve complex techniques, such as thin-film deposition.	2022 [41]
	Power consumption	- Designed for low power consumption. - Ideal for continuous monitoring applications.	- Active signal conditioning and amplification may increase power consumption.	2020 [42]
Piezoelectric	Sensitivity	- High sensitivity. - Fast response times. - Suitable for dynamic pressure measurements.	- Challenging interface with standard electronic circuits due to specific signal requirements. - Limitations in measuring static pressure.	2021 [43]
	Range of measurement	- Suitable for high-pressure measurements. - Effective in handling dynamic pressure changes.	- Limitations in measuring low-pressure values due to noise and signal-to-noise ratio issues.	2017 [44]
	Mechanical durability	- Endures mechanical stress. - Maintains performance during dynamic pressure applications.	- Extreme mechanical deformation may cause fatigue and reduced sensitivity in piezoelectric sensors.	2022 [45]
	Fabrication complexity	- Made using diverse materials and approaches. - Enables customized designs.	- Intricate fabrication, particularly for achieving high sensitivity and accuracy in piezoelectric sensors.	2019 [46]
	Power consumption	- Energy-efficient due to electrical signal generation in response to mechanical pressure.	- Increased power consumption due to the need for an external power source for continuous monitoring of piezoelectric signals.	2023 [47]

**Table 2:** Comparative analysis of sensing performance metrics in the different types of flexible and stretchable pressure sensors.

Sensing Performance Metrics	Piezoresistive	Capacitive	Piezoelectric
Sensitivity	Moderate to high	Good for small variations	High for precision
Dynamic range	Broad coverage	Broad coverage	Broad coverage
Linearity	Generally good	Generally good	Generally good
Response time	Fast	Fast	Fast
Recovery time	Quick	Quick	Quick
Hysteresis	Occasional	May occur	Possible
Drift	Possible over time	Typical minimal	Minimal over time
Resolution	High	High	High
Cross-sensitivity	Low compared to others	Possible due to environmental factors	Possible due to external factors

**Table 3:** Analysis of design strategies for sensor configurations of flexible and stretchable pressure sensors.

Sensor Configurations	Configuration	Working Principle	Advantages	Limitations	Year [Reference]
Thin film sensors	Thin film layer of sensing material on a flexible substrate.	- Resistive. - Capacitive. - Piezoelectric mechanisms.	- High sensitivity. - Fast response times - Excellent conformity to curved surfaces.	- Drift. - Hysteresis. - Delamination due to mechanical deformation and environmental factors.	2022 [89]
Microstructure sensors	Microscale or nanoscale structures (micro-pillars, microchannels, nanowires) for pressure-induced deformations.	- Changes in microstructure dimensions or orientations. - Altering Electrical. - Mechanical Properties.	- High sensitivity. - Excellent spatial resolution for detecting localized pressure variations.	- Complex - Require specialized techniques, such as micro/nanofabrication.	2022 [90] 2022 [91]
Textile-based sensors	Textile-based sensors integrate directly into textiles. Sensors can be woven, embroidered, printed, or attached onto fabrics, seamlessly integrating with clothing.	- Employ conductive materials (conductive yarns or inks) for sensing elements.	- Comfortable. - Flexible. - Washable. - Suitable for wearable applications.	- Lower sensitivity. - Lower accuracy compared to other configurations.	2020 [92] 2023 [93]

**Table 4:** Analysis of fabrication techniques for flexible and stretchable pressure sensors.

**Fabrication Techniques**	**Advantages**	**Limitations**	**Year [Reference]**
Printing and deposition methods			
Screen printing	- Cost-effective - Large-scale production - Conductive inks compatible with various substrates	- High-resolution patterns - Uniformity - Intricate designs - Varying layer thickness - Sensor performance	2023 [94]
Inkjet printing	- Excellent resolution - Fine features - Intricate designs - Variety of inks (conductive and sensing) - High accuracy	- Ink formulation - Viscosity control - Substrate choice - Post-processing - Performance - Stability	2022 [89]
Aerosol jet printing	- High resolution - Complex patterns - Variety of materials (conductive inks, dielectrics) - Layer thickness control	- Ink formulation - Aerosol parameters - Stable deposition - Reliable - Post-processing - Adhesion - Mechanical properties	2022 [90]
Magnetron sputtering	- High uniformity - Precise control - Versatile materials - Conformal coatings	- High equipment cost - Complex setup - Limited substrate size - Contamination risk	2021 [95]
Vacuum evaporation	- High purity - Precise control - Thin films - Uniform coatings	- Limited film thickness - Slow deposition rate - Material wastage	2019 [96]
Spin coating	- Uniform thin films - Simple process - Cost-effective - High throughput	- Limited film thickness control - Substrate size constraints - Material wastage	2022 [97]
Spraying	- Fast deposition - Large-area coverage - Adaptability to irregular surfaces	- Lower resolution - Potential overspray - Surface roughness	2022 [98]
Immersion/dip coating	- Uniform coatings - Simple process - Cost-effective - Suitable for complex shapes	- Limited control over - coating thickness - Longer processing time - Potential dripping	2021 [37] 2019 [99]
Microfabrication and patterning			
Photolithography	- High resolution - Precise patterning - Scalability - Batch processing	- Mask alignment - Complex setup - Limited substrate thickness	2023 [100]
Soft lithography	- High flexibility - Low-cost - Versatile - Replicable microstructures	- Limited resolution - Material compatibility - Multi-step process	2015 [101]
Laser machining	- High precision - Non-contact - Versatile materials - Complex structures	- Limited resolution - Heat-affected zone - Material selection	2022 [91] 2023 [102]
Assembly and integration processes			
Transfer printing	- High precision - Versatile - Multiple materials - Complex structures	- Alignment - Material compatibility - Multiple transfer steps	2022 [103]
Direct integration Techniques	- Simplified process - Precise integration - Reduced steps - Customizable designs	- Alignment challenges - Material compatibility - Process complexity	2020 [92] 2023 [93]

**Table 5:** State-of-the-art applications of flexible and stretchable pressure sensors.

Sensing Mechanisms	Material	Substrate	Sensitivity	Response/ Recovery Time	Year [Reference]
Healthcare and biomedical applications					
Piezoresistive	CNT/PDMS CCF	CPCs	5.1 kPa^−1^ (<15 kPa) 1.88 kPa^−1^ (15–50 kPa) 0.16 kPa^−1^ (>50 kPa)	54/65 ms	2023 [70]
Piezoresistive	Graphene/SWNTs	Tissue paper	12.6 kPa^−1^ (0–0.6 kPa) 4.3 kPa^−1^ (0.6–60.4 kPa)	214/380 ms	2023 [93]
Piezoresistive	GF/PDMS	PI	0.695 kPa^−1^ (0–14,122 kPa)	83/166 ms	2023 [128]
Piezoresistive	CNTs/SNWF composite	Fabric	GF = 8 (0–5 Strain (%))	55/40 ms	2022 [69]
Piezoresistive	Ti_3_C_2_T_x_ MXene	PET	0.61 kPa^−1^	160 ms	2022 [89]
Piezoresistive	Graphene/melamine	Sponge	0.186 kPa^−1^ (<5 kPa)	255/182 ms	2022 [90]
Piezoresistive	CNTs	Copper	1150.9 kPa^−1^ (<50 Pa)	43/15 ms	2022 [91]
Piezoresistive	PU/Ag	PDMS	10.53 kPa^−1^ (0–5 kPa) 0.97 kPa^−1^ (6–15 kPa)	60/30 ms	2022 [129]
Piezoresistive	Porous graphene (PG)	PI	53.99 MPa^−1^ (0.3–1000 kPa)	-	2021 [105]
Piezoresistive	PPy-Cotton	Cotton pads	4.48 kPa^−1^	220/240 ms	2020 [92]
Piezoresistive	rGO	Tissue paper	0.1 kPa^−1^ (2–20 kPa)	120/60 ms	2017 [130]
Capacitive	Electrode: Gold Dielectric: Nanofiber PVDF nonwoven fabrics	PDMS	1.807 kPa^−1^	48/72 ms	2023 [24]
Capacitive	Electrode: LIG Dielectric: PI paper	TPU film	0.0771 kPa^−1^ (0–9.6 kPa)	83/108 ms	2023 [26]
Capacitive	Electrode: Silver Thermoplastic polyether urethane (TPU)–ionic liquid (ILD)-h-BN (Ionic film)	PET	261.4 kPa^−1^ (0.05–450 kPa)	15/23 ms	2023 [94]
Capacitive	Dielectric: MXene/TPU	Silicon	10.2 kPa^−1^ (0–8.6 kPa) 3.65 kPa^−1^ (8.6–100 kPa)	41/39 ms	2021 [131]
Capacitive	Electrode: VG Dielectric: PDMS	PDMS	6.04 kPa^−1^ (0–1 kPa) 0.69 kPa^−1^ (1–10 kPa)	115/122 ms	2023 [132]
Capacitive	Dielectric: PVDF Nylon textile and polyvinylidene fluoride	Nylon textile	33.5 kPa^−1^	27/52 ms	2023 [133]
Capacitive	Electrode: CNT/PDMS Dielectric: PDMS	-	0.18 kPa^−1^	52/56 ms	2023 [134]
Capacitive	Electrode: Graphene/PET Dielectric: P(VDF-TrFE-CFE) [EMI] [TFSA]	-	10.14 kPa^−1^ (20 kPa)	<30/23 ms	2023 [135]
Capacitive	Dielectric: MWCNT/PDMS	-	2.155 kPa^−1^ (0–500 kPa)	90/248 ms	2023 [136]
Capacitive	Graphene−PVAc nanofibers	Aluminum foil	0.014 kPa^−1^ (2.73–56.06 kPa) 0.006 kPa^−1^ (75.76–318.18 kPa)	400/460 ms	2023 [137]
Piezoelectric	Ba_0_._94_Sr_0_._06_Sn_0_._09_Ti_0_._91_O_3_/PDMS Composite		21.09 mV⋅kPa^−1^	-	2023 [104]
Piezoelectric	PVDF/MXene-PVDF/ZnO (PM/PZ) nanofiber membranes	PU film	DL-PMPZ, I-PMPZ, CS-PM/PZ, and CS-PZ/PM sensors (0.626, 0.518, 0.628 and 0.751 V/N, respectively)	-	2023 [138]
Piezoelectric	BTO@PVDF piezoelectric	-	116 mV kPa^−1^ (0–10 N) 6.6 mV kPa^−1^ (10–100 N)	82.7 ms	2023 [139]
Piezoresistive, Piezoelectric	Porous wood (PW) and NaOH/Na_2_SO_3_ (H_2_O_2_)	-	0.443 V kPa^−1^ (0.06–0.6 kPa)	160/240 ms	2023 [140]
Human–machine interfaces and robotics applications					
Piezoresistive	MWCNT/PDMS	PDMS	0.7, 1.0, 1.3 kPa^−1^ (0–200 kPa)	12.5/37.5 ms	2023 [141]
Piezoresistive	AAO-PUA (bhAAO)	PUA	0.0275, 0.0653 kPa^−1^ (0.2 kPa)	352.3/177.2 ms	2023 [142]
Piezoresistive	PDMS/MWCNTs	PET	0.172 kPa^−1^	98.2/111.4 ms	2022 [128]
Piezoresistive	AgNWs/PDMS	PDMS	2.588 kPa^−1^	10/20 ms	2022 [143]
Piezoresistive	Au-PDMS	Au-PDMS	2.54 kPa^−1^	30/40 ms	2022 [144]
Piezoresistive	CNT/PDMS	PEN	50 kPa^−1^	24/32 ms	2020 [145]
Piezoresistive	CNT/PPy-PDA-PFDS	Textile	101.6, 147.4 kPa^−1^	80/60 ms	2019 [146]
Piezoresistive	MWCNTs/CB/PDMS	Silicon	3.2 kPa^−1^	217/81 ms	2019 [147]
Piezoresistive	Ag-PDMS	m-PDMS	2.525 kPa^−1^ (312 kPa)	45/70 ms	2023 [148]
Piezoresistive	PDMS/Bilayer graphene	PDMS	0.122 ± 0.002 kPa^−1^ (0–5 kPa) 0.077 ± 0.002 kPa^−1^ (5–20 kPa)	70 ms	2021 [149]
Piezoresistive	Graphene/PEO	Silicone	6.33 kPa^−1^	59/22 ms	2021 [150]
Piezoresistive	PDMS/IL/Keratin	Copper	0.037 kPa^−1^ (0–10 kPa)	8/11 ms	2020 [151]
Capacitive	Electrode: Carbon black Dielectric: Silicone	PU foam	0.047 N^−1^ (0.78 air volume fraction)	35 ± 5 ms	2021 [152]
Piezoelectric	Atactic polystyrene (aPS)	PET	d_app_ = 12.9 ± 1.8 pC N^−1^ Young modulus = 47.7 kPa	-	2021 [112]
Internet of things (IoT) and smart systems applications					
Piezoresistive	MoS_2_	PDMS	866.89 MPa^−1^ (0.46 MPa)	-	2021 [97]
Piezoresistive	Reduced graphene oxide (rGO)	Cotton fabric	3.96 kPa^−1^ (0–36 kPa)	170 ms	2021 [120]
Piezoresistive	AgNWs, Copper electrodes	Cotton fabrics	3.24 × 10^5^ kPa^−1^ (0–10 kPa) 2.16 × 10^4^ kPa^−1^ (10–100 kPa)	32/24 ms	2020 [119]
Piezoresistive	MXene	PET	99.5 kPa^−1^	4/13 ms	2020 [121]
Capacitive	Electrode: Conductive silicone rubber Dielectric: PDMS	-	0.12 kPa^−1^	21/36 ms	2023 [25]
Capacitive	Electrode: Cu MXene/HrGO hybrid film	PDMS	∼173,688 kPa^−1^	12/25 ms	2023 [124]

## Data Availability

Not applicable.
